# Older Adults’ User Engagement With Mobile Health: A Systematic Review of Qualitative and Mixed-Methods Studies

**DOI:** 10.1093/geroni/igad007

**Published:** 2023-01-30

**Authors:** Justine van Acker, Laura Maenhout, Sofie Compernolle

**Affiliations:** Faculty of Medicine and Health Sciences, Ghent University, Ghent, Belgium; Department of Movement and Sport Sciences, Faculty of Medicine and Health Sciences, Ghent University, Ghent, Belgium; Research Foundation Flanders (FWO), Brussels, Belgium; Department of Movement and Sport Sciences, Faculty of Medicine and Health Sciences, Ghent University, Ghent, Belgium; Research Foundation Flanders (FWO), Brussels, Belgium

**Keywords:** Digital health, Electronic health interventions, User experiences

## Abstract

**Background and Objectives:**

The aging population places increasing demands on our healthcare system. Mobile health offers the potential to reduce this burden. The aim of this systematic review is to thematically synthesize qualitative evidence of older adults’ user engagement toward mobile health, and to generate relevant recommendations for intervention developers.

**Research Design and Methods:**

A systematic literature search was performed in Medline, Embase, and Web of Science electronic databases from inception until February 2021. Papers on qualitative and mixed-methods studies that investigated older adults’ user engagement with a mobile health intervention were included. Relevant data were extracted and analyzed using thematic analysis. The Critical Appraisal Skills Program Qualitative Checklist was used to assess the quality of the included studies.

**Results:**

Thirty-two articles were deemed eligible for inclusion in the review. Three overarching analytical themes emerged from the 25 descriptive themes generated by the line-by-line coding: the limited capabilities, the prerequisite of motivation, and the importance of social support.

**Discussion and Implications:**

Successful development and implementation of future mobile health intervention for older adults will be challenging given the physical and psychological limitations and motivational barriers that older adults experience. Design adaptations and well-thought-out blended alternatives (i.e., combining mobile health with face-to-face support) might be potential solutions to improve older adults’ user engagement with mobile health interventions.

**Translational Significance:** User engagement with mobile health interventions is often low in older adults due to insufficient physical and psychological capabilities and motivational barriers. Intervention developers should adapt the design of future mobile health interventions to the limited capabilities of older adults. They should consider including social support options to overcome technological issues and to increase motivation, and they should focus on developing blended alternatives (i.e., combining mobile health with face-to-face support) to optimize older adults’ user engagement with mobile health interventions.

## Background

Population aging is a global phenomenon. Demographic projections predict that the global number of adults over the age of 60 will double to 2 billion by 2050 (United Nations Department of Economic and Social Affairs, 2017). This doubling poses major societal challenges, including challenges related to personal health and health care (World Health Organization, 2017). Indeed, becoming older is characterized by a progressive decline in functional capacity and an increased incidence of chronic diseases, such as cardiovascular disease, cancer, and diabetes (Brady et al., 2014; Kart et al., 1992; Pawelec et al., 2014). A recent systematic review reported that two-thirds of older adults (aged 65 years and over) suffer from multimorbidity (i.e., the presence of two or more chronic medical conditions in an individual). Two out of five have three or more chronic conditions, and one out of eight has even five or more chronic conditions (Ofori-Asenso et al., 2019). It goes without saying that this high prevalence of multimorbidity puts considerable pressure on our healthcare system (Glynn et al., 2011).

Mobile health (mHealth) interventions, a subset of digital health (i.e., use of information and communication technologies to improve human health, healthcare services, and wellness for individuals and across populations) specifically dedicated to use mobile devices to perform medical and public health practices (Wong & Wang, 2019), may assist older adults in the prevention, early detection, and management of chronic diseases and long-term conditions (Steinhubl et al., 2015). By doing so, mHealth interventions may offer a solution to unburden the healthcare system (Steinhubl et al., 2013). With the steady increase of smartphone and tablet use, mHealth interventions have gained increasing popularity, including among older adults (Seifert & Vandelanotte, 2021). Recent systematic reviews summarized existing mHealth interventions aimed at the primary, secondary, and tertiary prevention of chronic diseases in older adults (Kampmeijer et al., 2016; Matthew-Maich et al., 2016; Schorr et al., 2021). Findings indicated that mHealth interventions indeed offer the potential as a suitable tool for the promotion of health behaviors, self-management of chronic diseases, and medication adherence (Wildenbos et al., 2018). However, previous research also showed that, despite the interest and intention of older adults to use mHealth interventions (Seifert & Vandelanotte, 2021), user engagement with mHealth interventions is often low and inconsistent among the target group (Hoque & Sorwar, 2017; Lee & Coughlin, 2015).

User engagement, which is a complex, multidimensional concept, has been widely studied from different perspectives. Whereas behavioral scientists generally conceptualize user engagement as the degree of usage, computer scientists traditionally consider user engagement as the subjective experience with the intervention (Perski et al., 2017). As both perspectives are valuable and necessary to comprehensively understand the concept of user engagement, Perski et al. recently merged both perspectives into the following integrative definition: “Engagement with digital behavior-change interventions (DBCIs) is (1) the extent (e.g., amount, frequency, duration, depth) of usage and (2) a subjective experience characterized by attention, interest and affect” (Perski et al., 2017). Understanding older adults’ user engagement with mHealth interventions is needed, as a direct link between user engagement and intervention effectiveness has been assumed (Donkin et al., 2011).

Up till now, a number of qualitative and mixed-methods studies have been conducted to investigate older adults’ user engagement with mHealth interventions. The results of these studies have not been synthesized. Nevertheless, synthesizing the results from qualitative and mixed-methods studies can provide rich information to enhance the development and implementation of impactful mHealth interventions for this target group. Consequently, the aim of the current systematic review was to thematically synthesize qualitative studies that explored older adults’ user engagement toward mHealth interventions.

## Method

This systematic review was conducted according to the Enhancing Transparency in Reporting the Synthesis of Qualitative Research statement (Tong et al., 2012; see Supplementary Table 1). The protocol for the systematic review was registered on the International Prospective Register of Systematic Reviews with registration number: CRD42022322390.

### Search Strategy and Study Selection

A comprehensive literature search was conducted in the following databases: Medline (Pubmed interface), Embase (Embase.com interface), and Web of Science from inception until February 2021. The search strategy was developed by the first author (J. van Acker) using the PICO (population, intervention, control, outcome) acronym, and reviewed by an experienced health sciences research librarian. The population of interest was older adults, the intervention included mHealth interventions, and the study outcome was user engagement. The search strategy was piloted in Medline prior to being applied to the other databases. An example of the complete Medline search strategy, which was limited to peer-reviewed English-language articles, is available in Supplementary Table 2. After running the search strategy in each database, records were imported into Endnote, and duplicates were removed using the appropriate function within Endnote. Subsequently, the remaining records were uploaded to the Rayyan screening application, and titles, abstracts, and full texts were screened by two reviewers independently (J. van Acker and S. Compernolle) against the inclusion and exclusion criteria. Any disagreement between the reviewers was solved by discussion. Studies were included if they were conducted in older adults with a mean/median age of 60 years or older (depending on what was presented), and if they evaluated the user engagement of an mHealth intervention using a qualitative or mixed-methods study. Given the lack of consensus on the concept of user engagement, it was decided to broadly define user engagement, and include all studies assessing acceptability, feasibility, attitude, engagement, experience, satisfaction, and preferences toward/with mHealth interventions. mHealth interventions were defined as those interventions that use mobile communication technologies such as smartphones, personal digital assistants, hand-held tablets, or other portable and wireless devices to support medical and/or public health practice. Web-based interventions were only included if specifically stated that they were consulted using mobile devices. Interventions delivered using non-mobile devices were excluded, as well as studies using virtual reality and robots. Also, studies focusing on teleconsultations via video or telephone conversations were excluded, because we were mainly interested in automated mHealth interventions with minimal human involvement. Lastly, it was decided to also exclude studies in which participants were exposed to the mHealth intervention for less than 1 week, as the authors argued that a minimal exposure duration is needed to provide valuable information on user engagement.

### Quality Assessment

The Critical Appraisal Skills Program Qualitative Checklist was used to appraise the methodological quality of the included studies (“Critical appraisal skills qualitative checklist,” 2013). The checklist consists of 10 items assessing the research aims, qualitative methodology, research design, recruitment strategy, data collection, reflexivity of the researchers, ethical issues, data analysis, findings, and significance of the study. Each item was rated as “Yes,” “Can’t tell,” or “No” by two independent reviewers (J. van Acker and S. Compernolle), and a sum score was calculated to decide whether the included studies were of high (meeting at least 8 of the 10 criteria), medium (meeting 5–7 criteria), or low quality (meeting 4 or less criteria). When different ratings were assigned to a study by the two reviewers, a third reviewer (L. Maenhout) was consulted.

### Data Synthesis and Analysis

First, relevant study and intervention information were extracted using a standardized data extraction form in Microsoft Excel by the first and last authors independently. Second, all included studies were imported into NVivo 12 software to thematically synthesize the results of each study as described by Thomas and Harden (2008). During the first stage of the thematic synthesis, inductive line-by-line coding was performed to reduce the relevant data into meaningful codes. In the second stage, the initial codes were abductively organized (Thompson, 2022) into descriptive themes using the conceptual model of user engagement by Perski et al. (2017) and using more unanticipated inductive themes. The conceptual model classifies potential factors contributing to user engagement with DBCIs into two categories: intervention factors and contextual factors. Intervention factors consist of content-related (e.g., behavior-change techniques [BCTs]) and delivery-related (e.g., design, guidance, and personalization) factors. Contextual factors refer to the setting in which the intervention is used and the population using it. The setting is divided into physical factors (e.g., access, location, and policy) and social factors (e.g., culture, norms, and social cues). The population consists of demographic (e.g., age and gender), physical (e.g., physique), and psychosocial (e.g., motivation and self-efficacy) factors. In the final stage, analytical themes were developed by identifying conceptual links across themes and to go beyond the findings of the primary studies. The thematic synthesis process was conducted by two independent reviewers (J. van Acker and S. Compernolle). A third reviewer (L. Maenhout) was involved in cases of discrepancy.

## Results

### Study Selection

**Figure 1.**
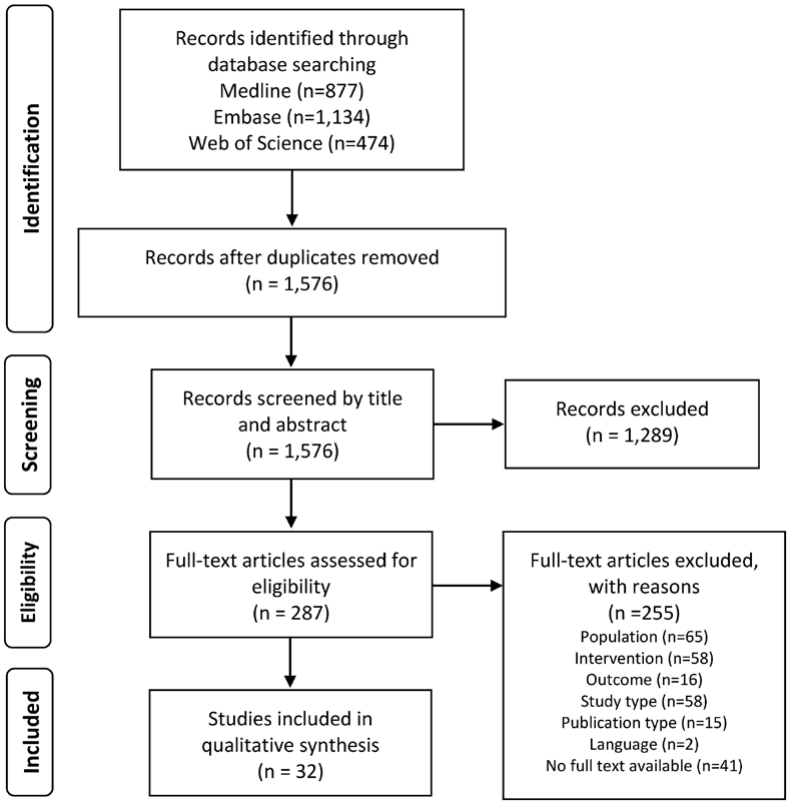
PRISMA flowchart of study selection process.

The study selection process is outlined using the PRISMA flowchart in Figure 1. A total of 2,485 records were identified, of which 909 were removed because they were duplicates. The titles and abstracts of the remaining 1,576 records were screened against the eligibility criteria, and 287 full texts were read. The final sample consisted of 32 papers.

### Study and Intervention Characteristics

Table 1 shows the study characteristics of the included studies. The majority of the included studies were conducted in Europe (*n* = 21). Six studies were conducted in North America, four in Australia, and one in Asia. Studies were published between 2014 and 2021. Almost half of the studies (14 of 32) used a mixed-methods design to explore older adults’ user engagement toward mHealth interventions. Eighteen studies used a qualitative design. Qualitative data were collected using focus groups (*n* = 6) and individual face-to-face interviews (*n* = 27) at the end of the intervention period. The sample size varied from 7 to 50 participants, and the mean age of the participants was between 60 and 86 years. The most frequently investigated outcomes were user experience and acceptability. Table 2 presents the intervention characteristics of the included studies. The majority of the interventions focused on primary (*n* = 13) or tertiary prevention (*n* = 18). Promoting physical activity was the most frequently occurring focus in primary prevention interventions (*n* = 9), whereas wheart failure self-care (*n* = 3) was most common in tertiary prevention interventions. A range of BCTs was deployed in the included interventions, and almost all interventions were delivered using a smartphone (*n* = 16) or a tablet (*n* = 18).

**Table 1.** Study Characteristics of the Included Studies

Study; Study Location	Study Design	Sample characteristics			Data Collection Method	Data Analysis	Outcome Variable
		Sample Size (% male)	Age: *M* (±*SD*) or Median and/or Range	Healthy vs Clinical Population			
Argent et al. (2019); Ireland	Mixed methods	15 (40%)	63.0 (±8.3) y	Knee replacement surgery patients	Individual semi-structured interviews	Thematic analysis with a grounded theory approach	Feasibility, usability, and user experiences
Arkkukangas et al. (2021); Sweden	Qualitative	12 (58%)	Mean age not defined; age range: 70–83	Community-dwelling older adults	Focus group interviews	Content analysis	User experiences
Bhattarai et al. (2020); Australia	Qualitative	16 (11%)	73.2 (±5.0) y	Older adults living with arthritic pain	Individual semi-structured interviews	Thematic analysis	Attitudes and experiences
Brickwood et al. (2020); Australia	Mixed methods	20 (40%)	73.6 (±5.5) y	Community-dwelling older adults (all included participants had at least one chronic condition)	Focus group interviews	Thematic analysis	Experiences and perceptions
Buck et al. (2017); United States	Qualitative	12 (27%)	67.0 y (±13.0)	Heart patients	Individual semi-structured interviews	Thematic analysis	Usability and experiences
Burkow et al. (2018); Norway	Mixed methods	10 (30%)	65.7 y (*SD* not defined)	COPD patients	Individual semi-structured interviews	Deductive thematic analysis and descriptive interpretation	Feasibility, acceptability, and usability
Cabrita et al. (2019); The Netherlands	Qualitative	12 (42%)	69.0 y (*SD* not defined)	Community-dwelling older adults	Individual semi-structured interviews	Inductive thematic analysis	Attitude and usability
Compernolle et al. (2020);	Mixed methods	28 (46%)	65.0 y (±4.6)	Community-dwelling older adults	Individual semi-structured interviews	Thematic analysis	Engagement, acceptability, and usability
Dithmer et al. (2016); Denmark	Mixed methods	10 (60%)	Mean age not defined; age range: 48–89	Heart patients	Individual semi-structured interviews	Content analysis	User experiences
Ehn et al. (2018); Sweden	Qualitative	8 (25%)	83.0 y (*SD* not defined)	Community-dwelling older adults	Individual semi-structured interviews	Inductive content analysis	User experiences and preferences
Gallagher et al. (2020); Australia	Mixed methods	12 (83%)	62.2 y (±5.3)	Coronary heart disease patients	Focus group interviews	Framework analysis	User experiences and preferences
Göransson et al. (2018); Sweden	Qualitative	17 (35%)	86.0 y (±6.5)	Older people with home-based health care	Individual semi-structured interviews	Inductive thematic analysis	User experiences
Hägglund et al. (2019); Sweden	Qualitative	17 (57%)	74.6 y (*SD* not defined)	Healthy community-dwelling older adults	Individual semi-structured interviews	Deductive content analysis	User experiences
Hawley-Hague et al. (2020); United Kingdom	Qualitative	7 (23%)	77.1 y (±8.5)	Older adults at risk of falls	Individual semi-structured interviews	Framework analysis	Usability and acceptability
Hill et al. (2018); United States	Qualitative	27 (42%)	79.0 y (±4.2)	Hip fracture surgery patients	Individual semi-structured interviews	Content analysis	Usability and acceptability
Jensen et al., 2019; Denmark	Qualitative	20 (35%)	78.0 y (*SD* not defined)	Hip fracture surgery patients	Individual semi-structured interviews	Content analysis	User experiences and usability
Kassavou et al. (2020); United Kingdom	Qualitative	22 (64%)	Mean age not defined; age range: 40–79	Patients with hypertension	Individual semi-structured interviews	Deductive thematic analysis	User experiences and acceptability
Kim et al. (2020); Korea	Mixed methods	11 (9%)	75.7 y (±3.9)	Dysphagia patients	Individual semi-structured interviews	Thematic analysis	Usability
Kim and Choudhury (2020); United States	Qualitative	15 (27%)	71.5 y (±6.3)	Community-dwelling older adults	Individual semi-structured interviews	Inductive and deductive grounded theory and thematic analysis	User experiences and attitudes
Lefler et al. (2018); United States	Mixed methods	28 (57%)	Mean age not defined	Heart failure patients	Individual semi-structured interviews	Content analysis	User experiences and feasibility
McBride et al. (2020); Ireland	Qualitative	11 (46%)	62.0 y (±9.1)	People with hypertension	Individual semi-structured interviews	Inductive thematic analysis	Usability and feasibility
Ogrin et al. (2018); Australia	Mixed methods	40 (N/A; sex not defined)	66.9 y (±17.1)	Diabetes patients	Individual semi-structured interviews and focus groups	Thematic analysis	Feasibility and acceptability
Paul et al. (2017); United Kingdom	Mixed methods	16 (50%)	71.1 y (±5.2)	Community-dwelling older adults	Focus group interviews	Thematic analysis using framework approach	Feasibility and acceptability
Peng et al. (2021); United States	Qualitative	50 (45%)	67.9 y (±2.0)	Community-dwelling older adults	Individual semi-structured interviews	Inductive and deductive thematic analysis	Feasibility and acceptability
Puri et al. (2017); Canada	Mixed methods	2 (40%)	64.0 y (*SD* not defined)	Community-dwelling older adults	Individual semi-structured interviews	Content analysis	Acceptance, usability, and user experiences
Roberts et al. (2019); United Kingdom	Qualitative	32 (69%)	60.0 y (*SD* not defined)	Cancer patients	Individual semi-structured interviews	Thematic analysis	User experiences
Scase et al. (2017); United Kingdom	Mixed methods	24 (8%)	75.2 y (±4.8)	Older adults with mild cognitive impairment	Focus group interviews	Thematic analysis	User experiences
Tabak et al. (2020); The Netherlands	Mixed methods	20 (50%)	71.0 y (±5.0)	Community-dwelling older adults	Individual semi-structured interviews	Not defined	Engagement, user experiences and usability
Tolstrup et al. (2020); Denmark	Mixed methods	14 (43%)	67.0 y (age range: 41–79)	Melanoma patients	Individual semi-structured interviews	Content analysis	User experiences
Torbjørnsen et al. (2019); Norway	Qualitative	24 (46%)	61.0 y	Type 2 diabetes patients	Individual semi-structured interviews	Content analysis	Acceptability and usability
Vailati Riboni et al. (2020); Italy	Qualitative	34 (29%)	72.0 y (±6.0)	Community-dwelling older adults	Individual semi-structured interviews	Thematic analysis	User experience and acceptance of technology
Williams et al. (2014); United Kingdom	Qualitative	19 (58%)	67.0 y (*SD* not defined)	COPD patients	Individual semi-structured interviews	Grounded theory approach	User experiences

*Notes*: COPD = chronic obstructive pulmonary disease; N/A = not applicable; *SD* = standard deviation.

**Table 2.** Intervention Characteristics of the Included Studies

Study	Description of the Intervention	Prevention Type	Content of the Intervention (BCTs)	Mode of Delivery
Argent et al., 2019	Exercise biofeedback system for orthopedic rehabilitation at home	Tertiary prevention	Provide feedback on performance Self-monitoring of behavior	A single wearable inertial measurement unit (Shimmer), and a tablet computer with an Android application
Arkkukangas et al., 2021	Mobile application for fall prevention exercise	Primary prevention	Provide instructions Self-monitoring of behavior	Otago Exercise Programme Android application on smartphone
Bhattarai et al., 2020	Pain management application for chronic arthritic patients	Tertiary prevention	Provide information on pain self-management Self-monitoring of pain	Rheumatoid Arthritis Information Support and Education (RAISE) application on smartphone or tablet
Brickwood et al., 2020	A wearable activity tracker and mobile application to maintain physical activity levels	Primary prevention	Provide feedback on performance Prompt specific goal setting Prompt review of behavioral goals Provide information	Jawbone UP24 activity tracker and associated application on mobile device
Buck et al., 2017	A heart failure self-care program	Secondary and tertiary prevention	Provide information Self-monitoring of behavior	A web-based, tablet-delivered Penn State Heart Assistant mHealth intervention
Burkow et al., 2018	A physical activity application in virtual groups for COPD	Tertiary prevention	Provide opportunities for social comparison Self-monitoring of behavior Instructions on how to perform a behavior Goal-setting behavior Problem solving Action planning Information about health consequences Demonstration of behavior Prompt/cues Rewards	A tablet-delivered Android application
Cabrita et al., 2019	Health behavior application	Primary prevention	Feedback on behavior Self-monitoring Goal reviewing	Fitbit Zip step counter, a smart scale Withings 30 and an Activity Coach application on the smartphone
Compernolle et al., 2020	A self-monitoring-based mHealth intervention to reduce sedentary behavior	Primary prevention	Feedback on behavior	Activator device and associated smartphone application
Dithmer et al., 2016	Gamification application to stimulate a healthy lifestyle in heart patients	Tertiary prevention	Social comparison Rewards	The Heart Game Android application on tablet
Ehn et al., 2018	Commercially available activity monitors to stimulate physical activity	Primary prevention	Feedback on performance Goal reviewing Self-monitoring Rewards	Two commercially available physical activity monitors: Withings Activité Pop (Withings) and Jawbone UP3 (Jawbone), together with corresponding application on tablet
Gallagher et al., 2020	Self-management application for coronary heart diseases	Secondary and tertiary prevention	Information on behavior Self-monitoring Prompts/cues Feedback on behavior	MyHeartMyLife application on tablet or smartphone
Göransson et al., 2018	General health and self-care application	Secondary prevention	Information on health behavior Self-monitoring	The Interaktor application on tablet or smartphone
Hägglund et al., 2019	Hearth failure self-care application	Secondary and tertiary prevention	Information on behavior Feedback on behavior Instruction on how to perform behavior Self-monitoring	mHealth system Optilogg (e.g., a tablet computer connected to a weight scale)
Hawley-Hague et al., 2020	Strength and balance home exercise smartphone applications to prevent falls	Primary prevention	Action planning Goal setting Feedback on behavior Prompts/cues Self-monitoring	“Motivate me” and “My Activity Programme” application and via the Samsung Galaxy S4 smartphone
Hill et al., 2018	Attention training application	Primary prevention	Not reported	Attention Training Application (ATA) on iOS tablet
Jensen et al., 2019	Empowerment application for self-care after hip fracture	Tertiary prevention	Information on behavior Instructions on how to perform a behavior	My Hip Fracture Journey application on tablet
Kassavou et al., 2020	Medication application for hypertension	Tertiary prevention	Information on health consequences and behavior Prompts/cues Feedback on behavior	Program on Adherence to Medication (PAM) SMS intervention and Android smartphone application
Kim et al., 2020	Swallowing training mHealth application	Tertiary prevention	Instructions on how to perform the behavior Self-monitoring behavior Feedback on behavior	365 Healthy Swallowing Coach application on tablet (Galaxy Tab A)
Kim & Choudhury, 2020	Activity trackers to support physical activity	Primary prevention	Not reported	A variety of activity tracking devices, including smartwatches, wristband-type activity trackers, and mobile applications for health care: Fitbit, iPhone Health application, H-Band, Weight Watchers application, My Fitness Pall application
Lefler et al., 2018	Self-care and heart failure management mHealth intervention	Secondary and tertiary prevention	Self-monitoring of behavior Feedback on behavior	Cloud DX Connected Health Kit containing an Android Health Tablet with Bluetooth-paired body weight scale and the Pulsewave Universal Serial Bus BP wrist monitor
McBride et al., 2020	Self-management and medication adherence in hypertension	Secondary and tertiary prevention	Self-monitoring Prompts/cues Feedback on behavior	BP Journal smartphone application and blood pressure monitor
Ogrin et al., 2018	Health education diabetes foot application	Tertiary prevention	Information on health consequences Instruction on how to perform behavior	Diabetes foot application on Android smartphone
Paul et al., 2017	Physical activity application	Primary prevention	Self-monitoring of behavior Feedback on behavior Goal setting Action planning Social support Social comparison Graded tasks Rewards Environmental restructuring	STARFISH application on smartphone
Peng et al., 2021	Activity trackers to support physical activity	Primary prevention	Not reported	Wearable activity trackers: Fitbit, Garmin, Miband, cell phone
Puri et al., 2017	Activity trackers to support physical activity	Primary prevention	Self-monitoring of behavior Feedback on behavior	Wearable activity trackers: Xiaomi Mi Band and Microsoft Band and associated application on Motorola Moto E smartphone
Roberts et al., 2019	Physical activity support in breast, colorectal and prostate cancer patient	Tertiary prevention	Goal setting Self-monitoring of behavior Feedback on behavior Social comparison Prompts/cues Rewards Action planning Instruction on how to perform behavior Graded tasks	Physical activity applications on smartphone: (1) Human, (2) The Walk game, (3) the Johnson & Johnson Official 7 Minute Workout and (4) Gorilla Workout
Scase et al., 2017	Gamification to improve health and wellbeing in older adults with mild cognitive impairment	Tertiary prevention	Self-monitoring of behavior Feedback on behavior Social support Information on behavior	DOREMI game application on tablet
Tabak et al., 2020	Game-based physical activity application	Primary prevention	Self-monitoring of behavior Feedback on behavior Goal setting Rewards	Fitbit met ActivityCoach application and WordFit game application on smartphone or tablet
Tolstrup et al., 2020	Side effects monitoring intervention during treatment with immunotherapy	Tertiary prevention	Self-monitoring of symptoms Prompt/cues	Electronic PRO tool on Ambuflex software platform on tablet
Torbjørnsen et al., 2019	Diabetes self-management application	Tertiary prevention	Providing information Self-monitoring Goal setting	A diabetes diary on an application (the Few Touch application) on a smartphone (the HTC HD Mini Windows Mobile 6.5) along with a OneTouch Ultra Easy LifeScan Inc. blood glucose measurement tool
Vailati Riboni et al., 2020	Mindfulness application	Primary prevention	Information on behavior Instructions on how to perform behavior	Mindfulness application on smartphone
Williams et al., 2014	COPD self-management application	Secondary and tertiary prevention	Information behavior Self-monitoring on behavior Feedback on behavior	EDGE application on tablet

*Notes*: BCT = behavior-change technique; COPD = chronic obstructive pulmonary disease.

### Quality Appraisal

The overall quality of the included studies was rated as “high” (*n* = 25), “medium” (*n* = 7), and “low” (*n* = 1) using the Critical Appraisal Skills Program checklist. The majority of the criteria were positively rated, except the criterion regarding the relationships, that is, the relationship between the researcher and the participants was rarely discussed. For details on the quality assessment, see Supplementary Table 3.

### Descriptive Themes

**Figure 2.**
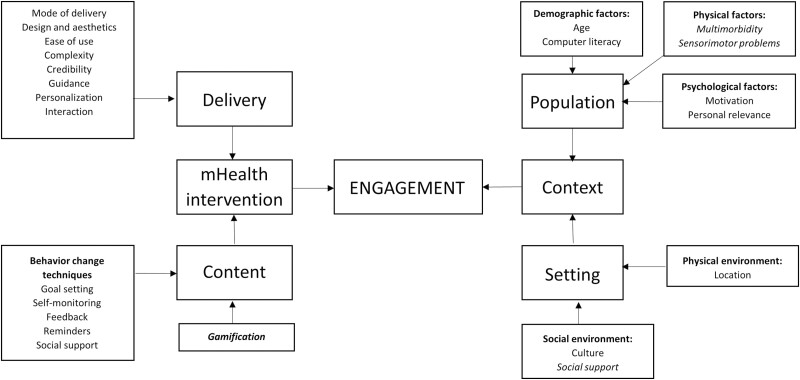
Descriptive themes based on the conceptual model of user engagement of Perski et al. (2017). Italicized factors are those that were not present in the initial model of Perski et al.

Twenty-five descriptive themes were identified based on the conceptual model of user engagement by Perski et al. (2017) and are presented in Figure 2. Descriptive themes were subdivided into contextual factors and characteristics of the mHealth intervention itself. Contextual factors included population characteristics (e.g., age, computer literacy, multimorbidity, sensorimotor problems, motivation, and personal relevance) and setting characteristics (e.g., culture, social support, and location). mHealth characteristics included factors related to the delivery (e.g., mode of delivery, design and esthetics, ease of use, complexity, credibility, guidance, personalization, interaction, and novelty) and factors related to the content of the intervention (e.g., goal setting, self-monitoring, feedback, reminders, social support, and gamification). Regarding the contextual factors, three new factors were identified, namely multimorbidity, sensorimotor problems, and social support. Regarding the mHealth characteristics, gamification was added to the content-related factors of Perski’s conceptual model.

### Analytical Themes

Three overarching analytical themes emerged from the descriptive themes generated by the line-by-line coding: (a) limited capabilities, (b) prerequisite of motivation, and (c) importance of social support. Table 3 provides an overview of the analytical themes, with corresponding descriptive themes and examples of quotations retrieved from the primary studies.

**Table 3.** Overview of Analytical Themes With Corresponding Descriptive Themes and Original Quotes

Analytical Themes	Descriptive Themes	Quotes
Limited capabilities	Physique	“Hearing somewhat difficult. Had to turn up sound to max PLUS hearing aids to max” (Hägglund et al., 2019)
	Computer literacy	“Very frustrating, demoralizing because I didn’t understand it. I wasn’t scoring anything, so I knew that there was something wrong, but didn’t know what. I felt awfully stupid” (Hill et al., 2018) “…I know with computers you can lose everything. I mean if I lose something, I have no idea how to find it. Or, if I change a setting and I can’t find or go to where I want it to go anymore so that’s why it is intimidating for me” (Kim & Choudhury, 2020) “To tell you the truth, I was afraid to use it just in case I broke it because I didn’t know anything about it” (Puri et al., 2017)
	Personal relevance	“[Human] was, as I say, very easy. It doesn’t cause you any difficulties or problems. So I think anybody can use it. You know, it doesn’t really matter how fit you are or how unfit you are, it’s not going to be a problem...[with Gorilla Workout] I found, even on the easy level, that some of the exercises were impossible...Level 1 is you can perform 0-10 push-ups, but they still kind of think you’re gonna be able to do some. It’s, like, I can’t do any. And I don’t think I’m ever gonna be” (Roberts et al., 2019) “I have quite a lot of arthritis in my ﬁngers…and gripping things is quite difficult for me. So there was a lot of information on that part of the app about putting a rubber band around a lid” (Bhattarai et al., 2020)
	Mode of delivery	“‘You’re probably better to have that app on a tablet… Arms are not long enough and the screen’s too small and it’s very hard” (Ogrin et al., 2018)
	Ease of use	“Only one thing maybe about the size and whatever, you know normally during the day it could sit in my pocket, but when you are in joggies and a T-shirt, I started to look for a T-shirt with a pocket. If it would be on a belt it would be a lot easier you know because I tended to leave it somewhere else” (Paul et al., 2017) “…At first, it was difficult to navigate through it because there is like, you press this, you get this menu and then you get this menu and then you get if you wanted to enter information, then you have to do all these things. But now I learned all and feel comfortable with using it” (Kim & Choudhury, 2020)
	Complexity	“I find in life, it’s called the KISS principle...keep it straight and simple...and the app in my opinion is easy to use...you have to make it easy to use...remember your clients that you are giving it to are not savvy...” (McBride et al., 2020) “..the bottom line is that...[The Walk’s] not intuitive...Perhaps I should have looked for a help area, or something, if I wanted to make full use of it, but then I also think, if an app is gonna be good, then it, it needs to lend itself to the user...with Human, again, I didn’t look out for any help areas. It’s just, you start using it, it tells you what, what’s going on, what you’ve done, and you can interpret it quite easily” (Roberts et al., 2019)
	Personalization	“… it can’t just be, like you said, a generic thing. Because people aren’t generic” (Bhattarai et al., 2020) “Everybody who’s had cancer will have a different level of fitness anyway even after cancer, and they’ll have a different level of motivation and a different starting point so that’s why that 7 minute app is good...you can choose...depending on where your starting point is” (Roberts et al., 2019)
	Aesthetics/design	“Well. I wouldn’t wear them out for the evening…Not if I was going out—depending on where I’m going, but they’re definitely not formal wear. [Anita] If it looked more like jewelry, I think more people would wear it. [Paula] No [aesthetics don’t matter]. I don’t see it as a fashion thing. [Greg]” (Puri et al., 2017) “The design of the app was very clear” (Compernolle et al., 2020)
Prerequisite of motivation	Motivation	“It is motivating to see in the evening that your percentage sitting time is high” (Compernolle et al., 2020) “It was motivating […] You sort of felt that you had to make a greater effort when you had that [tablet] there, you know. I thought it was great” (Burkow et al., 2018)
	Personal relevance	“I suppose it depends what you’re trying to get out of it and, for me, it’s looking at trying to regain a level of fitness, because I’ve probably lost it over the last four months or so. And I see the Seven Minute Workout as the one that will specifically do that whereas, [Human] is just monitoring what I will tend to do anyway” (Roberts et al., 2019) “I have quite a lot of arthritis in my ﬁngers…and gripping things is quite difficult for me. So there was a lot of information on that part of the app about putting a rubber band around a lid” (Bhattarai et al., 2020) …whether it’s going to be rheumatoid arthritis, or whether it’s going to be something else… you need to be able to have a button that says, “select your relevant (sic, arthritis),” …is yours osteo? is it rheumatoid?… (Bhattarai et al., 2020)
	Goal setting	“I went out for a walk speciﬁcally or walk long enough to make sure to get my target. I did that pretty well just about every day which is a big step forward” (Paul et al., 2017) “I remember one day I was at 11,500 [steps], at ten or half past ten to walk up to the dining room upstairs three, four times to get my ﬁve hundred steps, I wouldn’t lose my 500 steps” (Paul et al., 2017) “I find the, you know, the completion of the steps quite satisfying...if I’ve got to the evening and I’m on, you know, nine thousand and something, I want to make sure I’ve got that to 10,000 if I walk up and down the stairs a few times, and then actually when you go over, you know, you do feel quite pleased with yourself...[Fitbit] would plot how many days you’d done, how many steps and what your average was for the week and what your average was for the month and that was quite rewarding, because you do feel like you are achieving something” (Roberts et al., 2019)
	Self-monitoring	“It is really helpful to see the time you spent sitting” (Compernolle et al., 2020) “I’ve never been the type to make notes in a journal and check measurements and diet and things like that. But it was easier to make notes using the phone” (Torbjørnsen et al., 2019)
	Feedback and goal review	“What was missing for me was, once I had gotten a little way into the program, how much I had done and how much I had left” (Arkkukangas et al., 2021) “It shouldn’t be a problem for the same information to pop up on the doctor’s screen. I need a little push” (Torbjørnsen et al., 2019)
	Social comparison	“…if you could check with other people because it’s not always necessarily a lot of people placed around you who’ve got it (arthritis). You need to be talking to other people from here, there or other places” (Bhattarai et al., 2020)
	Gamification	“It would have been nice if you could not earn hammers in the crossword puzzle. That it would be more a reward for the no. of steps you take” (Tabak et al., 2020)
Importance of social support	Support of relatives	“…My grandson gave it to me as a gift and I’ve had it about 2 or 3 years. He explained to me how it was working because he had one already and he thought that it would be a good idea for his old grandmother and his aunts to have one, so we all have one” (Kim & Choudhury, 2020) “…My husband shows some functions (of Fitbit) to me. Then, I understand, I operate, and I work with that. After a few hours or few days, however, I forget that, and I will again ask him if he shows me again. It was not that easy to be familiar with that” (Kim & Choudhury, 2020)
	Professional support	“I felt it was doing me a whole heap of good, like somebody was always looking over my shoulder and keeping me (on track)” (Brickwood et al., 2020) “It’s nice to know that you’ve got it... you’re being monitored you know, and it gives a sense, you know, this is all getting back to some central computer and it’s all being monitored. So somebody’s actually looking at how you are, without me actually going to the doctors, so you get that feeling that you’re being looked after” (Williams et al., 2014) “The cardiologist can be in your pocket all the time... they’ll be able to communicate, you know look, what’s the matter with you” ... then come along like ‘don’t panic, should be alright, change this, change that’” (Gallagher et al., 2020)
	Guidance	“...if somebody isn’t getting advice from a professional first and they’re just picking up an app and...wanted to get a bit more active and doing it at home, I think that something like this could be actually be quite risky” (Roberts et al., 2019)

#### Limited capabilities

Older adults’ indicated that they experience physical and psychological limitations to successfully engage in mHealth interventions. Sensorimotor problems were frequently mentioned as physical limitations (Bhattarai et al., 2020; Hawley-Hague et al., 2020; Hill et al., 2018). For example, visual and hearing impairments, which are common in older adults, make it challenging to use mHealth interventions. Consulting information on small smartphone screens or hearing smartphone notifications was mentioned as being extremely difficult (Bhattarai et al., 2020; Hill et al., 2018). Also, older adults with arthritis indicated that the lack of mobility in their fingers limits them from efficiently using mHealth applications (Bhattarai et al., 2020; Hawley-Hague et al., 2020). Consequently, tablet-based interventions with large keys and good sound quality are preferred by the majority of older adults (Bhattarai et al., 2020; Burkow et al., 2018; Gallagher et al., 2020; Göransson et al., 2018; Paul et al., 2017). Regarding psychological limitations, computer literacy was often cited as an important constraint for mHealth user engagement (Brickwood et al., 2020; Jensen et al., 2019; Kim & Choudhury, 2020). Although levels of computer literacy varied greatly between older adults (Gallagher et al., 2020), the results of the included studies clearly indicated that the low levels of computer literacy in older adults negatively influenced the adoption and usage of mHealth interventions. Importantly, the lack of computer skills resulted not only in the nonuse or wrong use of certain functionalities, but also in the emergence of negative feelings, such as low self-esteem, anxiety, and depression (Brickwood et al., 2020; Ehn et al., 2018; Gallagher et al., 2020; Göransson et al., 2018; Williams et al., 2014). As such, older adults indicated that minimizing the complexity and maximizing the ease of use should be a key priority to optimize user engagement with future mHealth interventions (Göransson et al., 2018; Hill et al., 2018; McBride et al., 2020; Paul et al., 2017; Puri et al., 2017). In the study of Roberts et al., participants reported that the extent to which the level of difficulty of an mHealth app was suitable for the users affected the perceived personal relevance, which in turn positively influenced user engagement (Roberts et al., 2019). Next to the difficulty level of the application, the results also showed that the content of the intervention should be tailored to the individual needs of the users (Bhattarai et al., 2020; Kassavou et al., 2020; Peng et al., 2021). For example, older adults emphasized the importance of individualized, realistic goals to increase user engagement in mHealth apps (Peng et al., 2021). Tailored interventions are more likely to be motivating, which immediately brings us to the next analytical theme.

#### Prerequisite of motivation

Motivation seemed to be an important prerequisite for older adults’ user engagement with an mHealth intervention. Older adults that are motivated to monitor or change their behavior and health outcomes, as well as to use digital tools reported a higher user engagement (Ehn et al., 2018; Hägglund et al., 2019; Peng et al., 2021). In general, secondary and tertiary prevention mHealth interventions were considered to be more motivating, compared to primary prevention mHealth interventions. Both personal relevance and perceived usefulness were mentioned as contributing factors for these differences (Peng et al., 2021; Roberts et al., 2019; Williams et al., 2014). Older adults who feel like they do not need to change their behavior, or who do not understand the importance of a healthy lifestyle and self-management of diseases are not motivated to use mHealth interventions. Similarly, older adults with a low level of computer literacy and self-efficacy toward technology use, and a lack of interest in digital tools or applications is usually not motivated to start using an mHealth intervention. For those older adults who are motivated to start using an mHealth intervention, intervention content and delivery are important to maintain motivation (Argent et al., 2019; Ehn et al., 2018; Göransson et al., 2018). Regarding the content, several behavior- change techniques were found to be motivating—at least if they were implemented “in a good way.” First, prompting self-monitoring of health behavior and health-related parameters positively influenced older adults’ user engagement (Bhattarai et al., 2020; Cabrita et al., 2019; Ehn et al., 2018; Lefler et al., 2018; Williams et al., 2014). Second, prompting goal setting and intention formation positively affected older adults’ user engagement (Ehn et al., 2018; Hawley-Hague et al., 2020; Jensen et al., 2019; Roberts et al., 2019). In the study of Peng et al., self-monitoring and goal setting were mentioned as being the most important BCTs to support older adults in becoming a long-term user (Peng et al., 2021). As mentioned previously, setting personal and realistic goals was necessary to avoid users becoming demotivated (Peng et al., 2021; Roberts et al., 2019). Third, prompting review of behavioral goals and providing feedback on performance also contributed to sustained motivation and increased user engagement (Brickwood et al., 2020; Cabrita et al., 2019; Ehn et al., 2018; Kassavou et al., 2020). Importantly, feedback should be formulated positively, and constructively, also when the progression is the modest (Ehn et al., 2018; Tabak et al., 2020). Finally, providing opportunities for social comparison popped up as an important BCT to stay motivated (see the next analytical theme; Bhattarai et al., 2020; Hägglund et al., 2019; Scase et al., 2017; Tabak et al., 2020). Next to the BCTs, gamification also deserved attention when discussing older adults’ user engagement in mHealth interventions. Whereas some older adults found the included gamification elements disruptive and inappropriate (Argent et al., 2019; Roberts et al., 2019), other older adults perceived it as pleasant and attractive (Tabak et al., 2020).

#### Importance of social support

Receiving social support from family and friends positively influenced older adults’ user engagement with an mHealth intervention (Burkow et al., 2018; Kim & Choudhury, 2020). More positive attitudes were found if older adults’ relatives showed commitment with the mHealth intervention, and increased usage was reported if older adults’ relatives offered help with the installation and (first) use of the mHealth intervention (Burkow et al., 2018; Dithmer et al., 2016; Gallagher et al., 2020; Hägglund et al., 2019; Tabak et al., 2020). In particular, the help from younger family members, such as children and grandchildren, was perceived to be valuable, as younger people generally have more digital expertise (Gallagher et al., 2020; Kim & Choudhury, 2020). Next to family and friends, the active involvement and guidance of health practitioners were also highlighted as an important contributor to older adults’ user engagement with an mHealth intervention (Bhattarai et al., 2020; Roberts et al., 2019). mHealth interventions with professional support were considered to be more credible and motivating, and mHealth interventions that were recommended or delivered by health practitioners with whom the older adults have a relationship of trust were more likely to be used (Bhattarai et al., 2020; Brickwood et al., 2020; Roberts et al., 2019). Also, mHealth interventions focusing on the tertiary prevention of diseases were regarded as being risky if they were implemented without professional support. Finally, the role of other older adults and patients seemed to influence older adults’ user engagement with mHealth interventions. Stories and testimonies from older adults and patients in comparable situations were highly appreciated, as well as the ability to compare their own behavior/performance with the behavior/performance of others (Bhattarai et al., 2020; Hägglund et al., 2019). Especially, those older adults with a competitive character indicated that the latter improved their user engagement with the mHealth intervention (Burkow et al., 2018; Dithmer et al., 2016; Paul et al., 2017).

## Discussion

mHealth interventions for older adults have received growing attention in the literature in recent years. In light of the increasing number of studies, this systematic review aimed to synthesize the available qualitative evidence on older adults’ user engagement with mHealth interventions. A total of 32 studies were detected in this review, of which the majority only evaluated one aspect of user engagement (e.g., the extent of usage or the subjective experience characterized by intention, affect, and interest). Also, the intervention duration in the mainstream of studies was rather low, making it difficult to formulate conclusions on long-term user engagement. The thematic synthesis revealed 25 descriptive themes, and 3 analytical themes: the limited capabilities of older adults, the prerequisite of motivation, and the importance of social support. These themes should be carefully taken into account to improve future mHealth interventions for older adults.

### Descriptive Themes

Descriptive themes were identified based on the conceptual model of user engagement by Perski et al. (2017). Although the model is quite comprehensive and highly detailed, some suggestions and concerns are outlined to further optimize the model based on the results of the current review. First, the position of computer literacy (i.e., basic computer knowledge and skills) in the conceptual model is debatable. Although our results showed that computer literacy is definitely worth mentioning in the model, computer literacy should not be classified as a demographic factor. As computer literacy has to do with “cognitive skills,” we recommend classifying computer literacy as a psychological factor. Second, sensorimotor problems and social support appear to strongly influence older adults’ user engagement with mHealth interventions. Although sensorimotor problems and social support can be accommodated in physical characteristics and social environment, respectively, explicitly mentioning these concepts in the model is recommended to underlie their importance. Third, our results indicate considerable overlap between content and delivery of mHealth interventions. For example, the concepts “challenge,” “complexity,” and “credibility” are not only related to the delivery of an intervention, but might also be linked to the content of the intervention (e.g., exercises might not be challenging enough or too challenging). Modifications might be appropriate to clarify these links in the model. Lastly, gamification was not present in the initial model of Perski et al. Gamification has gained popularity in the past years (Sardi et al., 2017), and emerged as an important determinant of older adults’ user engagement with mHealth interventions. It is thus recommended to also add this concept to the model of user engagement.

### Analytical Themes

The first analytical theme focused on the limited capabilities of older adults. These limited capabilities are twofold: First, a loss of sensorimotor functioning was expressed by older adults, and second, computer literacy popped up as a major impeding factor. Unfortunately, the loss of sensorimotor functioning is an inevitable consequence of human aging (Buckles, 1993; Seidler & Stelmach, 1995). A U.S. study estimated that about 21% of older adults suffers from visual impairment, such as macular degeneration and bifocal glasses (Pascolini & Mariotti, 2012). Also, a population-based study conducted in Australia showed that the incidence of hearing loss was 48% in adults aged between 60 and 69 years (Graydon et al., 2019). Finally, arthritis and other rheumatic conditions are reported to affect 60% of the U.S. population aged 65 years and over. Consequently, adapting mHealth interventions to these common physical problems, which were also reported in the MOLD-US framework (Wildenbos et al., 2018) that provides an overview of physical and functional aging barriers related to the usability of mHealth interventions in older adults is imperative. Next to the sensorimotor problems, the prevalence of computer literacy also deserves attention in older adults. According to the latest Digital Economy Outlook Report from the Organization for Economic Cooperation and Development, 63% of 55- to 74-year olds were connected to the Internet (Oh et al., 2021). Despite the sharp increase in the number of older Internet users over the last few years, our results indicated that older adults’ (perceived) computer skills are still low. Presumably, this is caused by the fact that computers and the Internet were not a part of their childhood and early adult development (Poynton, 2005). To assist older adults with low computer literacy, instructional videos and, if possible, human support (i.e., blended approach) are recommended (de Main et al., 2022; van Middelaar et al., 2018).

Not surprisingly, the key role of motivation, which is a proximal determinant of behavior and central to most behavior-change theories, also emerged as an analytical theme (Lens & Vansteenkiste, 2020; Ryan, 2012). Within their systematic review, Kampmeijer et al. concluded that the successful use of eHealth and mHealth tools in health promotion programs for older adults greatly depends on older adults’ motivation (Kampmeijer et al., 2016). Motivation to start using an mHealth intervention is largely influenced by population characteristics (e.g., computer literacy), as well as by the type of prevention the mHealth intervention was focused on. Older adults were generally more motivated to get started with a secondary and/or tertiary prevention intervention, compared to a primary prevention intervention. This can be explained by the fact that the expected health consequences of primary prevention interventions typically occur in the long term, resulting in less subjective value than the more immediate rewards produced by secondary or tertiary prevention interventions (Blackburn et al., 2012; Rachlin et al., 1991). Consequently, future mHealth interventions focusing on primary prevention could be reframed on short-term positive consequences that can be directly felt (e.g., pleasure) rather than on long-term health benefits (Maltagliati et al., 2022). In contrast to initial motivation, motivation to long-term user engagement with an mHealth intervention appears to be mainly influenced by intervention characteristics. Although limited knowledge is available on how mHealth interventions can affect motivation (Asbjørnsen et al., 2019), our results suggest that certain BCTs, such as goal setting and feedback, contribute to older adults’ long-term user engagement. Existing evidence also suggests that older adults who have a personal aim and reachable goals are more likely to pursue the targeted behavior change and are thus more likely to become a long-term user (Kampmeijer et al., 2016). Next to the role of BCTs, previous research also highlighted the importance of gamification and persuasive design techniques to stimulate motivation, and affect user engagement (Asbjørnsen et al., 2019; Johnson et al., 2016). However, within the current review, no clear evidence was found for superior persuasive design techniques, and the views regarding gamification were mixed.

Finally, the importance of social support was derived from the thematic synthesis. Indeed, human support can improve user engagement and reduce attrition in digital health interventions (Ritterband et al., 2009). Older adults are interested in mHealth interventions as long as the mHealth intervention does not replace usual care. A previous systematic review, summarizing influencing factors for the success and failure of eHealth interventions, stated that the lack of face-to-face communication was considered a key barrier for the success of eHealth interventions (Granja et al., 2018). Within this review, it was stated that eHealth is considered to be impersonal, and therefore undermines the face-to-face communication of usual care (Granja et al., 2018). Based on this information, blended interventions should be preferred over stand-alone mHealth interventions. Next to the support from healthcare professionals, older adults also stressed the added value of peer support to increase user engagement with an mHealth intervention. Peer support can be included in mHealth interventions in several ways. Some examples are feeds, direct text messaging, and (social media) group chats (Fortuna et al., 2019). Finally, involving relatives in mHealth interventions was highly valued. Consequently, (blended) intergenerational interventions seem promising.

### Strengths and Limitations

To our knowledge, this is the first systematic review synthesizing existing qualitative evidence regarding older adults’ user engagement with mHealth interventions. As older adults have specific needs and unique perceptions on mHealth, studying this target group in isolation is useful (Batsis et al., 2019). The review employed a rigorous methodology, including an extensive search strategy, and a thorough quality appraisal. The range of identified papers was broad, as both primary, secondary, and tertiary prevention interventions were included. The use of thematic synthesis enabled us to go beyond primary studies, and to generate new interpretations in a systematic and well-documented way. As key descriptive themes were reported across many different papers, our findings can be considered to be robust. Despite these strengths, some limitations should be recognized when interpreting the results. The first limitation relates to the eligibility criteria of included studies. Only peer-reviewed papers written in English were included. Also, studies from which the full text was not available were excluded. Consequently, important evidence may have been missed, and selection bias may have occurred. Also, studies were eligible if the mean age was above 60 years. As such, quotes and views from participants younger than 60 years may also be included in the review. Moreover, the quality of some included studies was not optimal. The majority of the studies paid too little attention to the impact of the relationship between the researcher and the participants. Also, the intervention period of some included studies was rather short, which makes it difficult to formulate statements of long-term user engagement. Future research might consider including a larger minimum intervention duration to solve this problem. Additionally, the concept of user engagement was broadly defined due to the heterogeneity of definitions used in the past. Therefore, overlap might have arisen with other concepts, such as user experience (i.e., the perceptions and responses of older adults that result from using the intervention), acceptance (i.e., how well older adults perceived the intervention and the extent to which the intervention met their needs), and usability (i.e., the extent to which the intervention could be used by other older adults to improve their health; Compernolle et al., 2020). A clear taxonomy is needed to minimize overlap in the future. Lastly, the majority of the included studies only address one part of user engagement. This significantly affects the generalizability across descriptive themes. We hope with this review to encourage researchers that evaluate user engagement of mHealth, or broader, digital health interventions, to shed light on both the extent of usage as well as on the subjective experience characterized by attention, interest, and affect.

## Conclusion

Thirty-two studies were analyzed using thematic synthesis to explore older adults’ user engagement toward mHealth interventions. Although older adults are open to mHealth interventions, results showed that the majority of older adults encounter various barriers to successfully engage in mHealth interventions. These barriers are related to limited physical (e.g., sensorimotor deficits) and psychological capabilities (e.g., computer literacy), as well as to a lack of motivation. Providing social support was highly valued by older adults to overcome technological issues and to increase motivation. Adapting future mHealth interventions to the limited capabilities of older adults, finding the most supportive combination of BCTs and persuasive design techniques, and developing blended alternatives should be the main goal of future mHealth intervention developers to ensure long-term user engagement.

## Supplementary Material

igad007_suppl_Supplementary_Material

## Data Availability

The list of articles included in this systematic review is shared in the references and in the tables. The protocol for the systematic review was registered on the International Prospective Register of Systematic Reviews (PROSPERO) with registration number: CRD42022322390. Search terms and processes are shown in Supplementary Table 2 and in PROSPERO.
