# Supplementary material for: Older Adults’ User Engagement With Mobile Health: A Systematic Review of Qualitative and Mixed-Methods Studies
Source: Innov Aging. 2023 Jan 30;7(2):igad007. doi: 10.1093/geroni/igad007 (PMC10053647; doi:10.1093/geroni/igad007)
Supplement: igad007_suppl_Supplementary_Material [file igad007_suppl_supplementary_material.docx]

*Innovation in Aging* Online Supplementary Material: Justine Van Acker, Laura Maenhout, & Sofie Compernolle. Older adults’ user engagement with mobile health: A systematic review of qualitative and mixed methods studies.

Supplementary Table 1: ENTREQ checklist

| **No** | **Item** | **Guide and description** | **Page** |
| --- | --- | --- | --- |
| 1 | Aim | State the research question the synthesis addresses. | 4 |
| 2 | Synthesis methodology | Identify the synthesis methodology or theoretical framework which underpins the synthesis, and describe the rationale for choice of methodology (e.g., meta-ethnography, thematic synthesis, critical interpretive synthesis, grounded theory synthesis, realist synthesis, meta-aggregation, meta-study, framework synthesis). | 6 |
| 3 | Approach to searching | Indicate whether the search was pre-planned (comprehensive search strategies to seek all available studies) or iterative (to seek all available concepts until they theoretical saturation is achieved). | 4 |
| 4 | Inclusion criteria | Specify the inclusion/exclusion criteria (e.g., in terms of population, language, year limits, type of publication, study type). | 5 |
| 5 | Data sources | Describe the information sources used (e.g., electronic databases (MEDLINE, EMBASE, CINAHL, PsycINFO, Econlit), grey literature databases (digital thesis, policy reports), relevant organisational websites, experts, information specialists, generic web searches (Google Scholar) hand searching, reference lists) and when the searches conducted; provide the rationale for using the data sources. | 4 |
| 6 | Electronic search strategy | Describe the literature search (e.g., provide electronic search strategies with population terms, clinical or health topic terms, experiential or social phenomena related terms, filters for qualitative research, and search limits). | 5 |
| 7 | Study screening methods | Describe the process of study screening and sifting (e.g., title, abstract and full text review, number of independent reviewers who screened studies). | 5 |
| 8 | Study characteristics | Present the characteristics of the included studies (e.g., year of publication, country, population, number of participants, data collection, methodology, analysis, research questions). | 7 |
| 9 | Study selection results | Identify the number of studies screened and provide reasons for study exclusion (e,g, for comprehensive searching, provide numbers of studies screened and reasons for exclusion indicated in a figure/flowchart; for iterative searching describe reasons for study exclusion and inclusion based on modifications t the research question and/or contribution to theory development). | 6-7 |
| 10 | Rationale for appraisal | Describe the rationale and approach used to appraise the included studies or selected findings (e.g., assessment of conduct (validity and robustness), assessment of reporting (transparency), assessment of content and utility of the findings). | 5-6 |
| 11 | Appraisal items | State the tools, frameworks and criteria used to appraise the studies or selected findings (e.g., Existing tools: CASP, QARI, COREQ, Mays and Pope [25]; reviewer developed tools; describe the domains assessed: research team, study design, data analysis and interpretations, reporting). | 5 |
| 12 | Appraisal proces | Indicate whether the appraisal was conducted independently by more than one reviewer and if consensus was required | 6 |
| 13 | Appraisal results | Present results of the quality assessment and indicate which articles, if any, were weighted/excluded based on the assessment and give the rationale. | 7 |
| 14 | Data extraction | Indicate which sections of the primary studies were analysed and how were the data extracted from the primary studies? (e.g., all text under the headings “results /conclusions” were extracted electronically and entered into a computer software). | 6 |
| 15 | Software | State the computer software used, if any | 6 |
| 16 | Number of reviewers | Identify who was involved in coding and analysis. | 6 |
| 17 | Coding | Describe the process for coding of data (e.g., line by line coding to search for concepts) | 6 |
| 18 | Study comparison | Describe how were comparisons made within and across studies (e.g., subsequent studies were coded into pre-existing concepts, and new concepts were created when deemed necessary). | 6 |
| 19 | Derivation of themes | Explain whether the process of deriving the themes or constructs was inductive or deductive | 6 |
| 20 | Quotations | Provide quotations from the primary studies to illustrate themes/constructs, and identify whether the quotations were participant quotations of the author’s interpretation. | Table 3 |
| 21 | Synthesis output | Present rich, compelling and useful results that go beyond a summary of the primary studies (e.g., new interpretation, models of evidence, conceptual models, analytical framework, development of a new theory or construct). | 8-11 |

Supplementary Table 2: Search Medline

| **Concept** | **Search stragey (Medline)** |
| --- | --- |
| **1 - Older adults** | “aged” [Mesh] OR “aged” [TIAB] OR “elderly” [TIAB] OR “older adults” [TIAB] OR “older adult” [TIAB] OR “older patients” [TIAB] OR “older patient” [TIAB] OR “older people” [TIAB] OR “older users” [TIAB] OR “older user” [TIAB] OR “older subjects” [TIAB] OR “older subject” [TIAB] OR “seniors” [TIAB] OR “senior” [TIAB] OR “older woman” [TIAB] OR “older women” [TIAB] OR “older man” [TIAB] OR “older men” [TIAB] OR “older male” [TIAB] OR “older males” [TIAB] OR “older female” [TIAB] OR “older females” [TIAB] OR “older population” [TIAB] OR “older person” [TIAB] OR “older persons” [TIAB] |
| **2 - mHealth interventions** | “telemedicine” [Mesh] OR “telemedicine” [TIAB] OR “tele-medicine” [TIAB] OR “telehealth” [TIAB] OR “tele-health” [TIAB] OR “ehealth” [TIAB] OR “e-health” [TIAB] OR “electronic health” [TIAB] OR “mhealth” [TIAB] OR “m-health” [TIAB] OR “mobile health” [TIAB] OR “mobile technology” [TIAB] OR “mobile technologies” [TIAB] OR “mobile applications” [Mesh] OR “mobile applications” [TIAB] OR “mobile application” [TIAB] OR “health application” [TIAB] OR “health applications” [TIAB] OR “digital health” [TIAB] |
| **3 - User engagement** | “engagement“ [TIAB] OR “engaged“ [TIAB] OR “engaging” [TIAB] OR “engage” [TIAB] OR “attention” [Mesh] OR “attention” [TIAB] OR “usage” [TIAB] OR “usability” [TIAB] OR “usable” [TIAB] OR “useful” [TIAB] OR “usefulness” [TIAB] OR “experience” [TIAB] OR “experiences” [TIAB] OR “experiencing” [TIAB] OR “attractive” [TIAB] OR “attractiveness” [TIAB] OR “attractively” [TIAB] OR “attraction” [TIAB] OR “acceptability” [TIAB] OR “acceptance” [TIAB] OR “accept” [TIAB] OR “accepting” [TIAB] OR “attitude to computers” [Mesh] OR “attitude to computers” [TIAB] OR “adherence” [TIAB] OR “adhering” [TIAB] OR “patient satisfaction” [Mesh] OR “personal satisfaction” [Mesh] OR “satisfaction” [TIAB] OR “consumer behavior” [Mesh] OR “consumer behavior” [TIAB] OR “consumer behaviour” [TIAB] |
| **4 - Focus groups or interviews** | “interviews as topic” [Mesh] OR “narration” [Mesh] OR “narration” [TIAB] OR “narrative” [TIAB] OR “grounded theory” [Mesh] OR “grounded theory” [TIAB] OR “tape recording” [Mesh] OR “tape recording” [TIAB] OR “tape recordings” [TIAB] OR “focus groups” [Mesh] OR “focus group” [TIAB] OR “focus groups” [TIAB] OR “personal narratives as topic” [Mesh] OR “group discussion” [TIAB] OR “group discussions” [TIAB] OR “unstructured discussion” [TIAB] OR “unstructured discussions” [TIAB] OR “semi-structured discussion” [TIAB] OR “semi-structured discussions” [TIAB] OR “semistructured discussion” [TIAB] OR “semistructured discussions” [TIAB] OR “telephone questionnaire” [TIAB] OR “telephone questionnaires” [TIAB] OR “unstructured questionnaire” [TIAB] OR “unstructured questionnaires” [TIAB] OR “semi-structured questionnaire” [TIAB] OR “semi-structured questionnaires” [TIAB] OR “semistructured questionnaire” [TIAB] OR “semistructured questionnaires” [TIAB] OR “group interview” [TIAB] OR “group interviews” [TIAB] OR “telephone interview” [TIAB] OR “telephone interviews” [TIAB] OR “unstructured interview” [TIAB] OR “unstructured interview” [TIAB] OR “semi-structured interview” [TIAB] OR “semi-structured interview” [TIAB] OR “semistructured interview” [TIAB] OR “semistructured interview” [TIAB] OR “interview guide” [TIAB] OR “phenomenology” [TIAB] OR “ethnographic” [TIAB] OR “ethnography” [TIAB] OR “thematic analysis” [TIAB] OR “thematic analyses” [TIAB] OR “thematic framework” [TIAB] OR “content analysis” [TIAB] OR “content analyses” [TIAB] |
| **Combinations of concepts** | #1 AND #2 AND #3 AND #4 |

Supplementary Table 3: Quality assessment

| **Study** | **Aim** | **Methodology** | **Design** | **Recruitment** | **Data collection** | **Relationship** | **Ethiscal issues** | **Analysis** | **Results** | **Value** | **Global assessment** |
| --- | --- | --- | --- | --- | --- | --- | --- | --- | --- | --- | --- |
| Argent et al., 2019 | Yes | Yes | Yes | Yes | Yes | Can’t tell | Yes | No | Yes | Yes | High |
| Arkkukangas et al., 2021 | Yes | Yes | Yes | Yes | Yes | Can’t tell | Yes | Yes | Yes | Yes | High |
| Bhattarai et al., 2020 | Yes | Yes | Yes | Yes | Yes | No | Yes | Yes | Yes | Yes | High |
| Brickwood et al., 2020 | Yes | Yes | No | Can’t tell | Can’t tell | Yes | Yes | Yes | Yes | Can’t tell | Medium |
| Buck et al., 2017 | Yes | Yes | Yes | Yes | Yes | No | No | Yes | Yes | Yes | High |
| Burkow et al., 2018 | Yes | Yes | No | Can’t tell | Yes | Yes | Yes | Yes | Yes | Yes | High |
| Cabrita et al., 2019 | Yes | Yes | Yes | Yes | Yes | No | Yes | Can’t tell | Can’t tell | Yes | Medium |
| Compernolle et al., 2020 | Yes | Yes | Yes | Yes | Yes | No | Yes | Yes | Yes | Yes | High |
| Dithmer et al., 2016 | Yes | Yes | Yes | Can’t tell | Yes | No | Yes | No | Yes | Can’t tell | Medium |
| Ehn et al., 2018 | Yes | Yes | Yes | Yes | Yes | No | Yes | Yes | Yes | Yes | High |
| Gallagher et al., 2020 | Yes | Yes | Yes | Can’t tell | Yes | Can’t tell | Yes | Can’t tell | Yes | Yes | Medium |
| Göransson et al., 2018 | Yes | Yes | Yes | Yes | Yes | Yes | Yes | Yes | Yes | Yes | High |
| Hägglund et al., 2019 | Yes | Yes | Can’t tell | Yes | Yes | Can’t tell | Yes | Yes | Yes | Yes | High |
| Hawley-Hague et al., 2020 | Yes | Yes | Yes | Yes | Yes | No | Yes | Yes | Yes | Yes | High |
| Hill et al., 2018 | Yes | Yes | Yes | Yes | Yes | No | Yes | Yes | Yes | Yes | High |
| Jensen et al., 2019 | Yes | Yes | Yes | Yes | Yes | No | Yes | Yes | Yes | Yes | High |
| Kassavou et al., 2020 | Yes | Yes | Yes | Yes | Yes | No | Yes | Yes | Yes | Can’t tell | High |
| Kim et al., 2020 | Yes | Yes | Yes | Yes | Yes | No | Can’t tell | Yes | Yes | Yes | High |
| Kim & Choudhury, 2020 | Yes | Yes | Yes | Yes | Yes | No | No | Yes | Can’t tell | Yes | Medium |
| Lefler et al., 2018 | Yes | Yes | Yes | Yes | Yes | No | Can’t tell | Yes | Yes | Yes | High |
| McBride et al., 2020 | Yes | Yes | Yes | Yes | Yes | Yes | Yes | Yes | Yes | Yes | High |
| Ogrin et al., 2018 | Yes | Yes | Yes | Yes | Can’t tell | No | Yes | No | Can’t tell | Yes | Medium |
| Paul et al., 2017 | Yes | Yes | Yes | Can’t tell | Yes | No | Yes | Yes | Yes | Yes | High |
| Peng et al., 2021 | Yes | Yes | Yes | Yes | Yes | No | Can’t tell | Yes | Yes | Yes | High |
| Puri et al., 2017 | Yes | Yes | Yes | Yes | Yes | Can’t tell | Yes | Yes | Yes | Yes | High |
| Roberts et al., 2019 | Yes | Yes | Can’t tell | Yes | Yes | No | Yes | Yes | Yes | Yes | High |
| Scase et al., 2017 | Yes | Yes | Yes | Can’t tell | Can’t tell | No | No | Can’t tell | Yes | Can’t tell | Low |
| Tabak et al., 2020 | Yes | Yes | Yes | Yes | Can’t tell | No | Yes | Yes | Yes | Yes | High |
| Tolstrup et al., 2020 | Yes | Yes | Yes | Yes | Yes | No | Yes | Yes | Yes | Yes | High |
| Torbjørnsen et al., 2019 | Yes | Yes | Yes | Yes | Yes | No | Yes | Yes | Yes | Yes | High |
| Vailati Riboni et al., 2020 | Yes | Yes | Yes | Yes | Yes | No | Yes | Yes | Yes | Yes | High |
| Williams et al., 2014 | Yes | Yes | Yes | Yes | Yes | Yes | Yes | Yes | Yes | Yes | High |
